# Production of a chimeric flavivirus that contains the major structural glycoprotein genes of T’Ho virus in the genetic background of Zika virus

**DOI:** 10.1186/s12985-023-02172-2

**Published:** 2023-09-01

**Authors:** Chandra S. Tangudu, Alissa M. Hargett, Brooke C. Mitrisin, S. Viridiana Laredo-Tiscareño, Bradley J. Blitvich

**Affiliations:** Department of Veterinary Microbiology and Preventive Medicine, College of Veterinary Medicine, Iowa State University, Ames, IA 50011 USA

**Keywords:** Flavivirus, Orthoflavivirus, Zika virus, T’Ho virus, Chimeric, Differential diagnosis

## Abstract

T’Ho virus is a poorly characterized orthoflavivirus most closely related to Rocio virus and Ilheus virus, two orthoflaviviruses associated with human disease, suggesting that T’Ho virus could also be a human pathogen. The genome of T’Ho virus has been sequenced but an isolate has never been recovered, impeding its phenotypic characterization. In an attempt to generate recombinant T’Ho virus, the entire viral genome was synthesized as three overlapping DNA fragments, joined by Gibson assembly, and transfected into mosquito cells. Several cell culture passages were performed, but virus was not recovered. Subsequent experiments focused on the development of a chimeric orthoflavivirus that contains the premembrane and envelope protein genes of T’Ho virus in the genetic background of Zika virus. The chimeric virus replicated in mosquito (C6/36) and vertebrate (Vero) cells, demonstrating that the major structural glycoproteins of T’Ho virus permit entry into both cell types. The chimeric virus produced plaques in Vero cells that were significantly smaller than those produced by Zika virus. The chimeric virus can potentially be used as a surrogate diagnostic reagent in place of T’Ho virus in plaque reduction neutralization tests, allowing T’Ho virus to be considered in the differential diagnosis.

**Supplementary Information:**

The online version contains supplementary material available at 10.1186/s12985-023-02172-2.

The genus *Orthoflavivirus* (family *Flaviviridae*) is comprised of small, enveloped RNA viruses with single-stranded, positive-sense RNA genomes of ~ 11 kb. The genome contains a 5′ untranslated region (UTR) of about 100 nt., followed by a long open reading frame, and 3′ UTR of about 400–700 nt. [2, 17, 24]. The open reading frame encodes a large polyprotein that is proteolytically processed to generate three structural proteins, known as capsid (C), premembrane/membrane (prM/M), and envelope (E), and seven nonstructural proteins, known as NS1, NS2A, NS2B, NS3, NS4A, NS4B, and NS5. Multiple copies of the C protein encapsulate the genomic RNA, which together form the nucleocapsid. The nucleocapsid is surrounded by a host-derived lipid bilayer in which multiple copies of the E and M proteins are embedded. The E protein is responsible for receptor-mediated attachment and membrane fusion and is the primary target of neutralizing antibodies produced by the host during infection [35]. The prM protein is the precursor of the mature M protein and is required for the correct folding, maturation, and assembly of the E protein [26]. The nonstructural proteins have roles in viral genome replication, proteolytic processing of the polyprotein, virion assembly, and regulation of host immune responses [15].

T’Ho virus was discovered in mosquitoes in Mexico in 2007 and phylogenetic data revealed that its closest known relatives are Rocio virus (ROCV) and Ilheus virus (ILHV), two orthoflaviviruses associated with human disease [4, 11]. ROCV, a Biosafety Level-3 (BSL-3) agent, caused major outbreaks of encephalitis in Brazil in the 1970s, with a case fatality rate of 13% [10, 12, 28]. Permanent neurologic damage occurred in 20% of the patients who survived. ROCV was also responsible for several cases in Brazil in 2011–2013 [27]. ILHV is a BSL-2 agent distributed across much of Latin America and the Caribbean [8, 12, 20, 30]. Symptoms in humans usually present as fever, headache, and myalgia, but severe cases can progress to encephalitis and cardiac manifestations, sometimes with fatal outcomes. Because the closest known relatives of T’Ho virus are recognized human pathogens, T’Ho virus could be an unrecognized cause of human disease.

The genome of T’Ho virus has been sequenced in its entirety, but attempts to recover an isolate have been unsuccessful, impeding its phenotypic characterization [4, 11]. Without infectious virus, experimental infection studies cannot be performed to identify amplification vectors and reservoir hosts. Further, sera collected from humans and vertebrate animals during orthoflavivirus serological surveys cannot be tested for neutralizing antibodies to T’Ho virus because the plaque reduction neutralization test (PRNT) requires live virus [18]. In this study, our initial goal was to generate recombinant T’Ho virus, but these experiments were unsuccessful. Subsequent experiments focused on the development of a chimeric virus for potential use in PRNTs as a surrogate diagnostic reagent in place of T’Ho virus.

The chimeric genome was created by inserting the premembrane and envelope protein (prM-E) genes of T’Ho virus (strain T'Ho-Mex07) into the genetic background of Zika virus (ZIKV; strain PRVABC59). Four plasmids were required for these experiments: pUC19-THOVprME, pUC19-F1, pUC19-F2 and pUC19-F3 (Fig. 1). pUC19-THOVprME was created by synthetically generating the prM-E genes of T’Ho virus (genomic position 455-2458) as a dsDNA fragment (Bio-Basic Inc., Markham, ON, Canada) and blunt-end cloning the fragment into the Sma I restriction enzyme site of pUC19 (New England BioLabs, Ipswich MA). The three other plasmids contain the complete genome of ZIKV as overlapping DNA sequences and have been described elsewhere [31]. Briefly, pUC19-F1 contains a modified insect virus promoter, designated as OpIE2-CA, followed by ZIKV sequence (genomic position 1-3460) that spans the entire 5′ UTR through to the first 971 nt. of the NS1 gene. pUC19-F2 contains ZIKV sequence that spans the last 138 nt. of the NS1 gene through to the first 402 nt. of the NS5 gene (genomic position 3413-8071). pUC19-F3 contains ZIKV sequence that spans all of the NS5 gene, except for the first 344 nt., and all of the 3′ UTR (genomic position 8016-10,807), followed by the hepatitis delta virus anti-genomic ribozyme sequence (HDVr) and simian virus 40 polyadenylation signal (SV40p) [32].

**Fig. 1:**
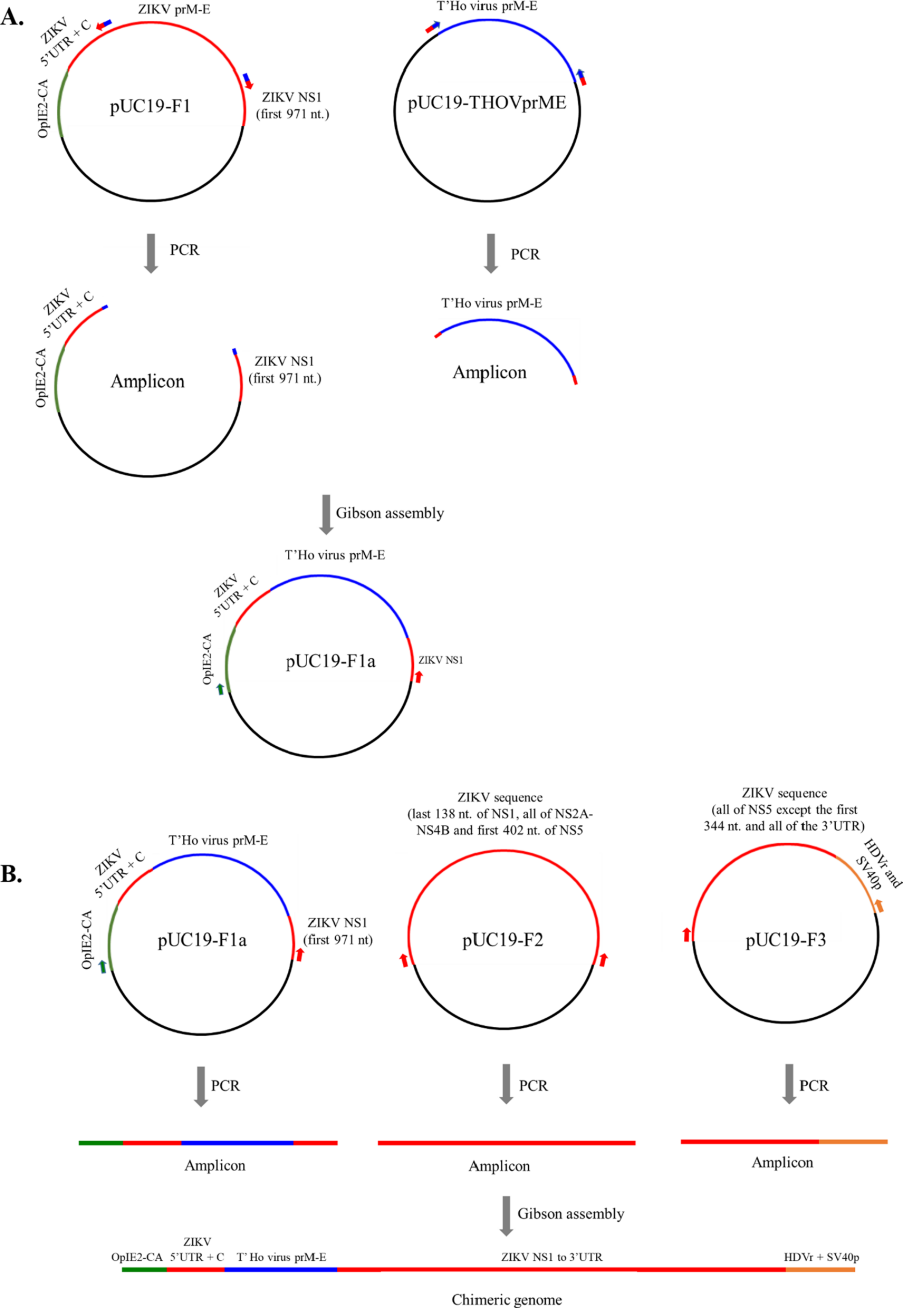
Schematic of the strategy used to create the chimeric genome of ZIKV/THOV(prM-E). **A** Construction of pUC19-F1a. Initial experiments required the use of two plasmids, designated pUC19-F1 and pUC19-THOVprME. pUC19-F1 contains an insect virus promoter (OpIE2-CA), followed by ZIKV sequence (ZIKV genomic position 1-3460) that spans all of the 5′ UTR through to the first 971 nt. of the NS1 gene. pUC19-THOVprME contains the prM-E gene sequences of T’Ho virus (T’Ho virus genomic position 455-2461). The aforementioned plasmids were used as templates in PCRs. One PCR amplified all of pUC19-F1, except for ZIKV prM-E. The reaction was performed using a forward chimeric primer specific to the 3′ and 5′ ends of the T’Ho virus E gene and ZIKV NS1 gene, respectively, and a reverse chimeric primer specific to the 3′ and 5′ ends of the ZIKV C gene and T’Ho prM gene, respectively. Another PCR was designed to amplify all of T’Ho virus prM-E from pUC19-THOVprME. The reaction was performed using a forward chimeric primer specific to the 3′ and 5′ ends of the ZIKV C gene and T’Ho virus prM gene, respectively and a reverse chimeric primer specific to the 3′ and 5′ ends of the T’Ho virus E gene and ZIKV NS1 gene, respectively. The two amplicons were joined by Gibson assembly, yielding a plasmid designated as pUC19-F1a. The newly created plasmid contains the prM-E sequences of T’Ho virus, flanked at the 5′ end by OpIE2-CA and the 5′ UTR and C sequences of ZIKV and flanked at the 3′ end by the first 971 nt. of the NS1 gene of ZIKV. **B** Generation of the full-length chimeric orthoflavivirus genome. Subsequent experiments required the use of pUC19-F1a and two additional plasmids, designated as pUC19-F2 and pUC19-F3. pUC19-F2 contains ZIKV sequence that spans the last 138 nt. of the NS1 gene through to the first 402 nt. of the NS5 gene (ZIKV genomic position 3413-8071). pUC19-F3 contains ZIKV sequence that spans all of the NS5 gene, except for the first 344 nt., and all of the 3′ UTR (ZIKV genomic position 8016-10,807), followed by the hepatitis delta virus anti-genomic ribozyme sequence (HDVr) and simian virus 40 polyadenylation signal (SV40p). The aforementioned plasmids were used as templates in PCRs that amplified all of the viral sequences and none of the cloning vector (pUC19) sequences. Primers were designed so that each amplicon contained an overlap of about 50 bp with the adjacent amplicon(s). The three amplicons were joined by Gibson assembly, yielding a linear chimeric orthoflavivirus genome flanked by OpIE2-CA sequence at its 5′ end and HDVr and SV40p sequences at its 3′ end. Sequences are color-coded: pUC19 (black), OpIE2-CA (green), ZIKV (red), T’Ho virus (blue) and HDVr/SV40p (orange). PCR primers are denoted as small arrows, which are color-coded according to the sequence to which they bind. Chimeric primers are represented by bicolored arrows

pUC19-F1 was modified by replacing the prM-E sequences of ZIKV with the corresponding sequences of T’Ho virus (Fig. 1A). To this end, a PCR was performed using pUC19-F1 as template, a forward chimeric primer specific to the 3′ and 5′ ends of the T’Ho virus E gene and ZIKV NS1 gene, respectively, and a reverse chimeric primer specific to the 3′ and 5′ ends of the ZIKV C gene and T’Ho prM gene, respectively. The resulting amplicon encompassed all of pUC19-F1, except for the prM-E sequences of ZIKV. Another PCR was performed using pUC19-THOVprME as template and a forward chimeric primer specific to the 3′ and 5′ ends of the ZIKV C gene and T’Ho virus prM gene, respectively and a reverse chimeric primer specific to the 3′ and 5′ ends of the T’Ho virus E gene and ZIKV NS1 gene, respectively. The resulting amplicon contained the prM-E sequences of T’Ho virus. The chimeric primers used in these PCRs were designed so that the two amplicons contained overlapping sequences of approximately 50 bp at each terminus. The amplicons were joined by Gibson assembly using established protocols [5] to yield a plasmid designated as pUC19-F1a. The newly created plasmid was transformed into chemically competent *E. coli* cells and colonies that contained viral sequences with no mutations were identified.

Additional PCRs were performed using pUC19-F1a, pUC19-F2, and pUC19-F3 as templates and chimeric primers that amplified all of the viral sequences, but none of the pUC19 sequences, from each plasmid (Fig. 1B). The chimeric primers were designed so that each amplicon contained an overlap of about 50 bp with the adjacent amplicon(s). Amplicons were joined by Gibson assembly and the reaction products were analyzed by RT-PCR and Sanger sequencing using primers spanning the entire genome to ensure that there were no mutations. Assembled DNAs were transfected into *Aedes albopictus* (C6/36) cells using Lipofectamine™ 2000 Transfection Reagent (ThermoFisher Scientific). Transfected cells were incubated for 7 days then an aliquot of supernatant was inoculated onto new monolayers of C6/36 cells. A second passage was performed and lysates and supernatants were harvested from the final cell culture passage at 5 days post-inoculation (p.i.) and assayed for virus by Western blot and immunofluorescence assay (IFA), as previously described [29, 31]. A similar strategy was used in an attempt to generate recombinant T’Ho virus. Briefly, the entire genome of T’Ho virus, including the UTRs, was synthesized as three overlapping DNA fragments and blunt-end cloned into pUC19 (Bio-Basic Inc.). OpIE2-CA sequence was located immediately upstream of the ZIKV 5′ UTR and HDVr and SV40p sequences were located immediately downstream of the ZIKV 3′ UTR. Viral sequences were amplified from the plasmids by PCR and the resulting amplicons were joined by Gibson assembly and sequenced. Reaction products were transfected into C6/36 cells, but recombinant T’Ho virus was not recovered (data not shown).

Chimeric virus was successfully recovered and designated as ZIKV/THOV(prM-E). Western blot analysis revealed that ZIKV/THOV(prM-E) replicates in both mosquito (C6/36) and vertebrate (Vero) cells (Figs. 2A and 3A, respectively; Additional file 1). The Western blot analysis was performed using polyclonal antibodies that react with ZIKV C, prM, and NS1, and cellular β-actin. ZIKV C and NS1 antigens were detected in C6/36 and Vero cells inoculated with ZIKV/THOV(prM-E). In contrast, antigen was not detected in C6/36 or Vero cells when the anti-ZIKV prM antibody was used, consistent with chimeric virus containing the prM gene of T’Ho virus. The positive and negative controls yielded expected results. ZIKV C, prM, and NS1 antigens detected in ZIKV-inoculated, but not mock-inoculated, C6/36 and Vero cells. The intensities of the β-actin bands were similar, indicating that there was approximately equal loading of samples in each lane.

**Fig. 2:**
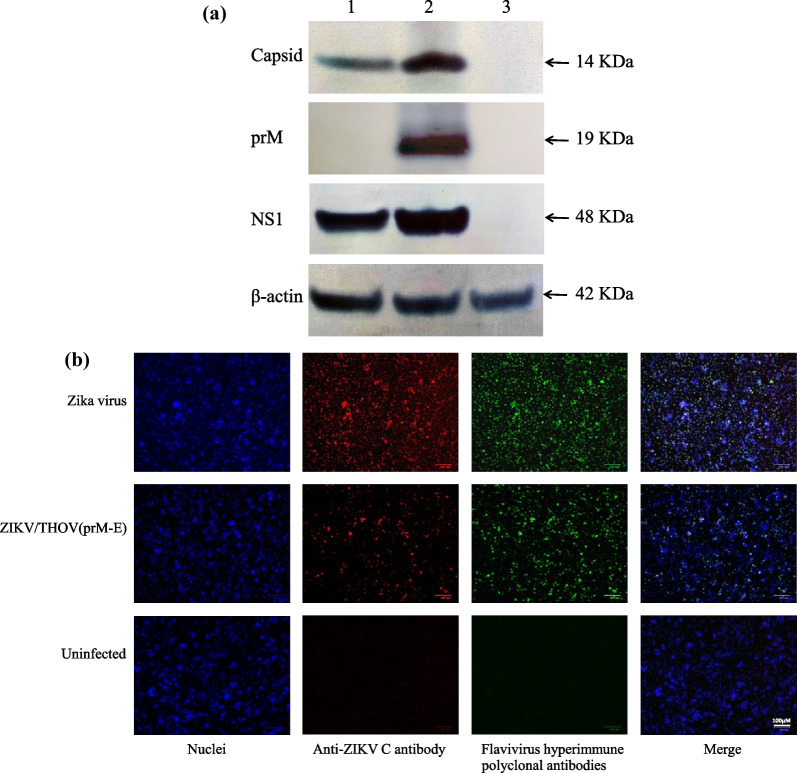
Mosquito cells support the replication of ZIKV/THOV(prM-E). **A** Western blot analysis of C6/36 cells inoculated with ZIKV/THOV(prM-E). C6/36 cells approaching confluency in 25 cm^2^ culture flasks were inoculated with ZIKV/THOV(prM-E) or ZIKV at a multiplicity of infection of 0.1 or they were inoculated with media only (lane 1–3, respectively). The chimeric virus had undergone two consecutive passages in C6/36 cells prior to the experiment. Lysates were harvested at 5 days p.i. then equal amounts of protein were resolved on 8–16% Tris–glycine gels and analyzed by Western blot using (i) anti-ZIKV C polyclonal antibody, (ii) anti-ZIKV prM polyclonal antibody, (iii) anti-ZIKV NS1 polyclonal antibody or (iv) anti-β-actin polyclonal antibody. The arrows show the expected migration positions of ZIKV C and NS1 (molecular weights: 12, 19 and 48 KDa, respectively) and cellular β-actin (molecular weight: 42 KDa). **B** IFA analysis of C6/36 cells inoculated with ZIKV/THOV(prM-E). C6/36 cells approaching confluency in 35 mm^2^-well culture dishes were inoculated with ZIKV/THOV(prM-E), ZIKV or media only (rows 1–3, respectively). The chimeric virus had undergone two consecutive passages in C6/36 cells prior to the experiment. Cells were fixed with methanol at 5 days p.i. and immunostained with an anti-ZIKV C polyclonal antibody (column 2) or a pooled suspension of heterologous hyperimmune polyclonal antibodies to ZIKV and several other orthoflaviviruses (column 3), followed by a pooled suspension of Alexa Fluor 594-conjugated donkey anti-rabbit IgG and Alexa Fluor 488-conjugated goat anti-mouse IgG. DAPI was used to visualize the nucleic (column 1). Merged images are also shown (column 4). A magnification of 3,500X was used. Scale: 100 µM

**Fig. 3:**
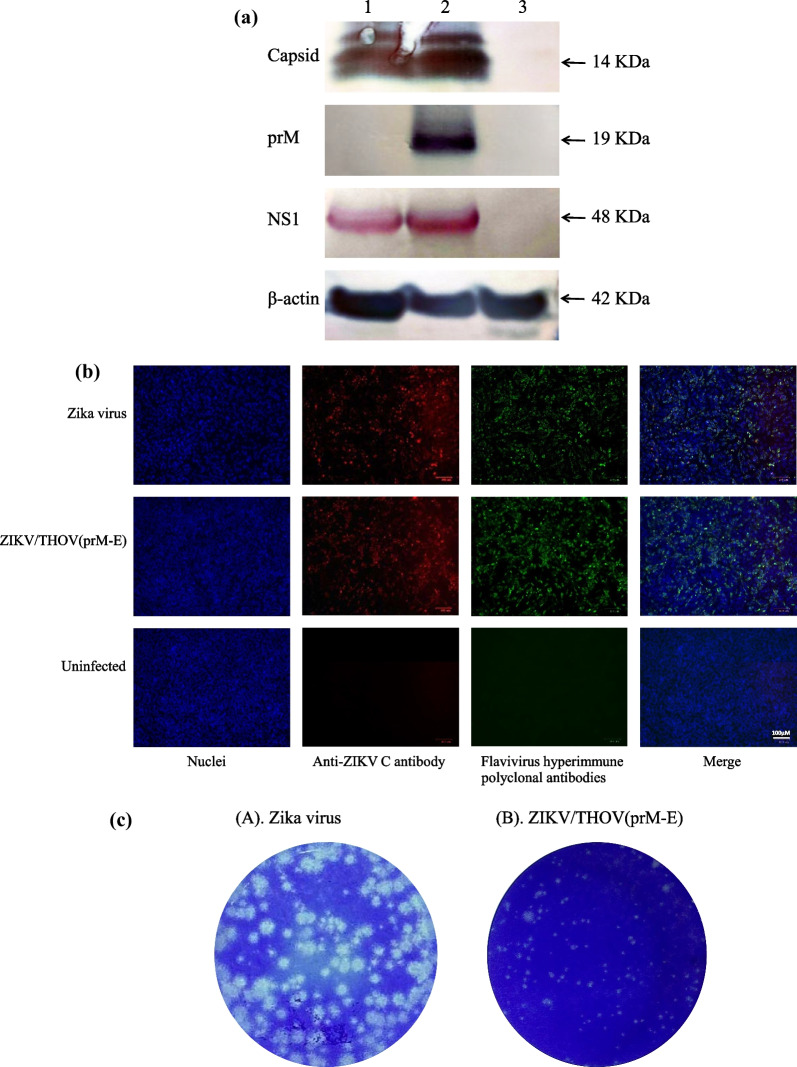
Vertebrate cells support the replication of ZIKV/THOV(prM-E). **A** Western blot analysis of Vero cells inoculated with ZIKV/THOV(prM-E). Vero cells approaching confluency in 25 cm^2^ culture flasks were inoculated with ZIKV/THOV(prM-E) or ZIKV at a multiplicity of infection of 0.1 or they were inoculated with media only (lane 1–3, respectively). The chimeric virus had undergone two consecutive passages in Vero cells prior to the experiment. Lysates were harvested at 3 days p.i. then equal amounts of protein were resolved on 8–16% Tris–glycine gels and analyzed by Western blot using (i) anti-ZIKV C polyclonal antibody, (ii) anti-ZIKV prM polyclonal antibody, (iii) anti-ZIKV NS1 polyclonal antibody or iv) anti-β-actin polyclonal antibody to ensure there was approximately equal loading in each lane. The arrows show the expected migration positions of ZIKV C, prM, and NS1 (molecular weights: 12, 19 and 48 KDa, respectively) and cellular β-actin (molecular weight: 42 KDa). **B** IFA analysis of Vero cells inoculated with ZIKV/THOV(prM-E). Vero cells approaching confluency in 35 mm^2^-well culture dishes were inoculated with ZIKV/THOV(prM-E), ZIKV or media only (rows 1–3, respectively). The chimeric virus had undergone two consecutive passages in Vero cells prior to the experiment. Cells were fixed with methanol at 3 days p.i. and immunostained with an anti-ZIKV C polyclonal antibody (column 2) or a pooled suspension of heterologous hyperimmune polyclonal antibodies to ZIKV and several other orthoflaviviruses (column 3), followed by a pooled suspension of Alexa Fluor 594-conjugated donkey anti-rabbit IgG and Alexa Fluor 488-conjugated goat anti-mouse IgG. DAPI was used to visualize the nucleic (column 1). Merged images are also shown (column 4). A magnification of 3500X was used. Scale: 100 µM. **C** Comparison of the plaque morphologies of ZIKV/THOV(prM-E) and ZIKV. Confluent monolayers of Vero cells in 35 mm^2^ culture dishes were inoculated with (A) ZIKV/THOV(prM-E) or (B) ZIKV then incubated for 5 days and fixed. Two replicate experiments were performed and at least 40 plaques were measured for each virus

IFA analysis confirmed that ZIKV/THOV(prM-E) replicates in C6/36 and Vero cells (Figs. 2B and 3B, respectively). These experiments were performed using rabbit anti-ZIKV C polyclonal antibody and a pooled suspension of hyperimmune polyclonal antibodies from mice inoculated with ZIKV and several other orthoflaviviruses. Capsid antigen was detected in C6/36 and Vero cells inoculated with ZIKV/THOV(prM-E) and ZIKV. The heterologous orthoflavivirus hyperimmune polyclonal antibodies recognized antigen in C6/36 and Vero cells inoculated with ZIKV/THOV(prM-E) and ZIKV. Viral antigen was not detected in mock-inoculated C6/36 and Vero cells.

We compared the sizes of plaques produced by ZIKV/THOV(prM-E) and ZIKV in Vero cells at 5 days p.i. (Fig. 3C). The chimeric virus produced plaques with a mean diameter ± 1 standard deviation of 1.08 ± 0.48 (95% CI) mm. ZIKV plaques had a mean diameter ± 1 standard deviation of 3.18 ± 0.84 (95% CI) mm. A statistical analysis revealed that the difference in plaque sizes is significant (t-test *p* < 0.0001). For each virus, 40 plaques were measured per experiment, with duplicate experiments performed.

ZIKV/THOV(prM-E) could potentially be used as a diagnostic tool. Researchers performing orthoflavivirus serosurveillance in Mexico and elsewhere in the Americas could include the chimeric virus in PRNTs, allowing T’Ho virus to be considered in the differential diagnosis. Other chimeric orthoflaviviruses have been developed for use in PRNTs [13, 16, 25]. These chimeric viruses were developed as surrogates for orthoflaviviruses currently or previously classified as BSL-3 agents (Japanese encephalitis virus, St. Louis encephalitis virus and West Nile virus) or that grow relatively slowly or produce small plaques (dengue viruses 1–4). The chimeric viruses were created by inserting the prM-E genes of the orthoflavivirus of interest into genetic backbone of the live-attenuated yellow fever virus vaccine [13, 16, 25]. Chimeric alphaviruses have also been developed for use as diagnostic tools, allowing PRNTs to be performed under BSL-2 conditions when testing for antibodies to Eastern and Venezuelan equine encephalitis viruses, which are BSL-3 agents [14, 21].

One limitation of our study is that the diagnostic efficacy of ZIKV/THOV(prM-E) was not compared to T’Ho virus by PRNT. All of the other chimeric flaviviruses and alphaviruses mentioned earlier were compared to the parental viruses that contributed the immunogenic structural protein genes and shown to be suitable surrogates in PRNTs [13, 14, 16, 21, 25]. The unavailability of an isolate of T’Ho virus clearly prevents us from performing this comparison. However, there is no other virus that can be used in PRNTs in place of ZIKV/THOV(prM-E). Because no other diagnostic tools are currently available, researchers performing orthoflavivirus serosurveillance in the Americas should consider including ZIKV/THOV(prM-E) in their PRNTs.

ZIKV/THOV(prM-E) requires BSL-2 containment because it was built on the genetic background of a BSL-2 agent. T’Ho virus is not listed in the Biosafety in Microbiological and Biomedical Laboratories but its closest known relative, Rocio virus, is a BSL-3 pathogen, suggesting that T’Ho virus should be considered a BSL-3 pathogen [19]. Therefore, if an isolate of T’Ho virus is eventually recovered, those without access to BSL-3 facilities may not be permitted to work with it. Many research and diagnostic laboratories lack access to BSL-3 facilities, highlighting the important need for a surrogate virus that can be used in PRNTs under BSL-2 containment.

The ability of ZIKV/THOV(prM-E) to replicate in C6/36 and Vero cells demonstrates that the major structural glycoproteins of T’Ho virus permit entry into both mosquito and vertebrate cells. However, experiments designed to determine whether T’Ho virus replicates in these cells cannot be performed unless an isolate is acquired or recombinant virus is produced. At present, the arthropod and vertebrate host ranges of T’Ho can only be inferred using information obtained for its closest known relatives. ROCV cycles in nature between birds and *Psorophora ferox* mosquitoes [28]. The major reservoir hosts and amplification vectors of ILHV are birds and arboreal mosquitoes of multiple genera [7, 22, 33].

ZIKV/THOV(prM-E) plaques were significantly smaller than ZIKV plaques. Chimeric orthoflaviviruses created from prM-E gene exchanges often produce plaques smaller than at least one parental virus [3, 6, 23, 34]. For example, a chimeric virus that contained the prM-E genes of dengue virus 2 in the genetic background of dengue virus 4 produced plaques significantly smaller than those of both parental viruses [3]. The plaque morphologies of ZIKV/THOV(prM-E) could not be compared to both parental viruses because an isolate of T’Ho virus is unavailable.

Attempts to generate recombinant THo virus were unsuccessful. One explanation for this outcome is the viral genome sequence deposited into the Genbank database contains sequence errors. The full genome of T’Ho virus was sequenced by unbiased high-throughput sequencing, except for the terminal ends where 5′ and 3′ rapid amplification of cDNA ends and Sanger sequencing were used [4]. The majority of the unbiased high-throughput sequencing data were confirmed by Sanger sequencing, but there was an insufficient amount of sample to verify the authenticity of the entire genomic sequence. Another explanation is that T’Ho virus cannot replicate in C6/36 and Vero cells. This explanation is unlikely because the closest known relatives of T’Ho virus replicate in these cell types and these cell lines are commonly used for arbovirus propagation [1, 9].

To conclude, we report the construction and characterization of ZIKV/THOV(prM-E), a chimeric orthoflavivirus that contains the prM-E genes of T’Ho virus in the genetic background of ZIKV. The ability of the chimeric virus to replicate in C6/36 and Vero cells provides evidence that the major structural glycoproteins of T’Ho virus permit entry into both mosquito and vertebrate cells. ZIKV/THOV(prM-E) could provide a suitable surrogate for T’Ho virus in PRNTs. Unfortunately, the diagnostic efficiencies of ZIKV/THOV(prM-E) and T’Ho virus could not be compared because infectious T’Ho virus is not available. However, there is no other virus than can be used in place of ZIKV/THOV(prM-E) and therefore, researchers performing orthoflavivirus serosurveillance in the Americas may want to include it in their PRNTs.

### Supplementary Information

**Additional file 1.** **Figure S1.** Mosquito and vertebrate cells support the replication of ZIKV/THOV(prM-E). **A** Western blot analysis of C/36 cells inoculated with ZIKV/THOV(prM-E). These are the same images as shown in Figure 2A, except they are uncropped. Experimental details are provided in the legend for Figure 2A. **B** Western blot analysis of Vero cells inoculated with ZIKV/THOV(prM-E). These are the same images as shown in Figure 3A, except they are uncropped. Experimental details can provided in the legend for Figure 3A

## Data Availability

The datasets used and/or analysed during the current study are available from the corresponding author on reasonable request.

## References

[1] Amarilla AA, Fumagalli MJ, Figueiredo ML, Lima-Junior DS, Santos-Junior NN, Alfonso HL, Lippi V, Trabuco AC, Lauretti F, Muller VD, Colon DF, Luiz JPM, Suhrbier A, Setoh YX, Khromykh AA, Figueiredo LTM, Aquino VH (2018). Ilheus and Saint Louis encephalitis viruses elicit cross-protection against a lethal Rocio virus challenge in mice. PLoS ONE.

[2] Barrows NJ, Campos RK, Liao KC, Prasanth KR, Soto-Acosta R, Yeh SC, Schott-Lerner G, Pompon J, Sessions OM, Bradrick SS, Garcia-Blanco MA (2018). Biochemistry and molecular biology of flaviviruses. Chem Rev.

[3] Bray M, Lai CJ (1991). Construction of intertypic chimeric dengue viruses by substitution of structural protein genes. Proc Natl Acad Sci USA.

[4] Briese T, Lorono-Pino MA, Garcia-Rejon JE, Farfan-Ale JA, Machain-Williams C, Dorman KS, Lipkin WI, Blitvich BJ (2017). Complete genome sequence of T'Ho virus, a novel putative flavivirus from the Yucatan Peninsula of Mexico. Virol J.

[5] Charles J, Tangudu CS, Nunez-Avellaneda D, Brault AC, Blitvich BJ (2021). The host range restriction of bat-associated no-known-vector flaviviruses occurs post-entry. J Gen Virol.

[6] Charlier N, Molenkamp R, Leyssen P, Paeshuyse J, Drosten C, Panning M, De Clercq E, Bredenbeek PJ, Neyts J (2004). Exchanging the yellow fever virus envelope proteins with Modoc virus prM and E proteins results in a chimeric virus that is neuroinvasive in SCID mice. J Virol.

[7] Cunha MS, Luchs A, da Costa AC, Ribeiro GO, Dos Santos FCP, Nogueira JS, Komninakis SV, Marinho R, Witkin SS, Villanova F, Deng X, Sabino EC, Delwart E, Leal E, Nogueira ML, Maiorka PC (2020). Detection and characterization of Ilheus and Iguape virus genomes in historical mosquito samples from Southern Brazil. Acta Trop.

[8] da Costa VG, Saivish MV, Lino NAB, Bittar C, de Freitas Calmon M, Nogueira ML, Rahal P (2022). Clinical landscape and rate of exposure to Ilheus virus: Insights from systematic review and meta-analysis. Viruses.

[9] de Souza Lopes O, Coimbra TL, de Abreu Sacchetta L, Calisher CH (1978). Emergence of a new arbovirus disease in Brazil. I. Isolation and characterization of the etiologic agent, Rocio virus. Am J Epidemiol.

[10] de Souza Lopes O, de Abreu Sacchetta L, Coimbra TL, Pinto GH, Glasser CM (1978). Emergence of a new arbovirus disease in Brazil. II. Epidemiologic studies on 1975 epidemic. Am J Epidemiol.

[11] Farfan-Ale JA, Lorono-Pino MA, Garcia-Rejon JE, Hovav E, Powers AM, Lin M, Dorman KS, Platt KB, Bartholomay LC, Soto V, Beaty BJ, Lanciotti RS, Blitvich BJ (2009). Detection of RNA from a novel West Nile-like virus and high prevalence of an insect-specific flavivirus in mosquitoes in the Yucatan Peninsula of Mexico. Am J Trop Med Hyg.

[12] Figueiredo LT (2000). The Brazilian flaviviruses. Microbes Infect.

[13] Johnson BW, Kosoy O, Hunsperger E, Beltran M, Delorey M, Guirakhoo F, Monath T (2009). Evaluation of chimeric Japanese encephalitis and dengue viruses for use in diagnostic plaque reduction neutralization tests. Clin Vaccine Immunol.

[14] Johnson BW, Kosoy O, Wang E, Delorey M, Russell B, Bowen RA, Weaver SC (2011). Use of sindbis/eastern equine encephalitis chimeric viruses in plaque reduction neutralization tests for arboviral disease diagnostics. Clin Vaccine Immunol.

[15] Knyazhanskaya E, Morais MC, Choi KH (2021). Flavivirus enzymes and their inhibitors. Enzymes.

[16] Komar N, Langevin S, Monath TP (2009). Use of a surrogate chimeric virus to detect West Nile virus-neutralizing antibodies in avian and equine sera. Clin Vaccine Immunol.

[17] Lindenbach BD, Randall G, Bartenschlager R, Rice C, Howley PM, Knipe DM, Whelan S (2020). *Flaviviridae*: the viruses and their replication. Fields Virology.

[18] Maeda A, Maeda J (2013). Review of diagnostic plaque reduction neutralization tests for flavivirus infection. Vet J.

[19] Meechan PJ, Potts J. Biosafety in microbiological and biomedical laboratories. 6th Edition. (2020). https://www.cdc.gov/labs/pdf/SF__19_308133-A_BMBL6_00-BOOK-WEB-final-3.pdf

[20] Milhim B, Estofolete CF, Rocha LCD, Liso E, Brienze VMS, Vasilakis N, Terzian ACB, Nogueira ML (2020). Fatal outcome of Ilheus virus in the cerebrospinal fluid of a patient diagnosed with encephalitis. Viruses.

[21] Ni H, Yun NE, Zacks MA, Weaver SC, Tesh RB, da Rosa AP, Powers AM, Frolov I, Paessler S (2007). Recombinant alphaviruses are safe and useful serological diagnostic tools. Am J Trop Med Hyg.

[22] Plante JA, Plante KS, Popov VL, Shinde DP, Widen SG, Buenemann M, Nogueira ML, Vasilakis N (2023). Morphologic and genetic characterization of Ilheus virus, a potential emergent flavivirus in the Americas. Viruses.

[23] Pletnev AG, Karganova GG, Dzhivanyan TI, Lashkevich VA, Bray M (2000). Chimeric Langat/Dengue viruses protect mice from heterologous challenge with the highly virulent strains of tick-borne encephalitis virus. Virology.

[24] Postler TS, Beer M, Blitvich BJ, Bukh J, de Lamballerie X, Drexler JF, Imrie A, Kapoor A, Karganova GG, Lemey P, Lohmann V, Simmonds P, Smith DB, Stapleton JT, Kuhn JH (2023). Renaming of the genus flavivirus to orthoflavivirus and extension of binomial species names within the family Flaviviridae. Arch Virol.

[25] Pugachev KV, Guirakhoo F, Mitchell F, Ocran SW, Parsons M, Johnson BW, Kosoy OL, Lanciotti RS, Roehrig JT, Trent DW, Monath TP (2004). Construction of yellow fever/St. Louis encephalitis chimeric virus and the use of chimeras as a diagnostic tool. Am J Trop Med Hyg.

[26] Roby JA, Setoh YX, Hall RA, Khromykh AA (2015). Post-translational regulation and modifications of flavivirus structural proteins. J Gen Virol.

[27] Saivish MV, da Costa VG, Rodrigues RL, Feres VCR, Montoya-Diaz E, Moreli ML (2020). Detection of Rocio virus SPH 34675 during Dengue Epidemics, Brazil, 2011–2013. Emerg Infect Dis.

[28] Saivish MV, Gomes da Costa V, de Lima Menezes G, Alves da Silva R, Dutra da Silva GC, Moreli ML, Sacchetto L, Pacca CC, Vasilakis N, Nogueira ML (2021). Rocio virus: an updated view on an elusive flavivirus. Viruses.

[29] Saiyasombat R, Carrillo-Tripp J, Miller WA, Bredenbeek PJ, Blitvich BJ (2014). Substitution of the premembrane and envelope protein genes of Modoc virus with the homologous sequences of West Nile virus generates a chimeric virus that replicates in vertebrate but not mosquito cells. Virol J.

[30] Spence L, Anderson CR, Downs WG (1962). Isolation of Ilheus virus from human beings in Trinidad, West Indies. Trans R Soc Trop Med Hyg.

[31] Tangudu CS, Charles J, Nunez-Avellaneda D, Hargett AM, Brault AC, Blitvich BJ (2021). Chimeric Zika viruses containing structural protein genes of insect-specific flaviviruses cannot replicate in vertebrate cells due to entry and post-translational restrictions. Virology.

[32] Varnavski AN, Young PR, Khromykh AA (2000). Stable high-level expression of heterologous genes in vitro and in vivo by noncytopathic DNA-based Kunjin virus replicon vectors. J Virol.

[33] Vieira C, Andrade CD, Kubiszeski JR, Silva D, Barreto ES, Massey AL, Canale GR, Bernardo CSS, Levi T, Peres CA, Bronzoni RVM (2019). Detection of Ilheus virus in mosquitoes from southeast Amazon, Brazil. Trans R Soc Trop Med Hyg.

[34] Wang HJ, Li XF, Ye Q, Li SH, Deng YQ, Zhao H, Xu YP, Ma J, Qin ED, Qin CF (2014). Recombinant chimeric Japanese encephalitis virus/tick-borne encephalitis virus is attenuated and protective in mice. Vaccine.

[35] Zhang X, Jia R, Shen H, Wang M, Yin Z, Cheng A (2017). Structures and functions of the envelope glycoprotein in flavivirus infections. Viruses.

