# Supplementary figures and images for: Production of a chimeric flavivirus that contains the major structural glycoprotein genes of T’Ho virus in the genetic background of Zika virus
Source: Virol J. 2023 Sep 1;20:197. doi: 10.1186/s12985-023-02172-2 (PMC10472631; doi:10.1186/s12985-023-02172-2)

Figure 2A

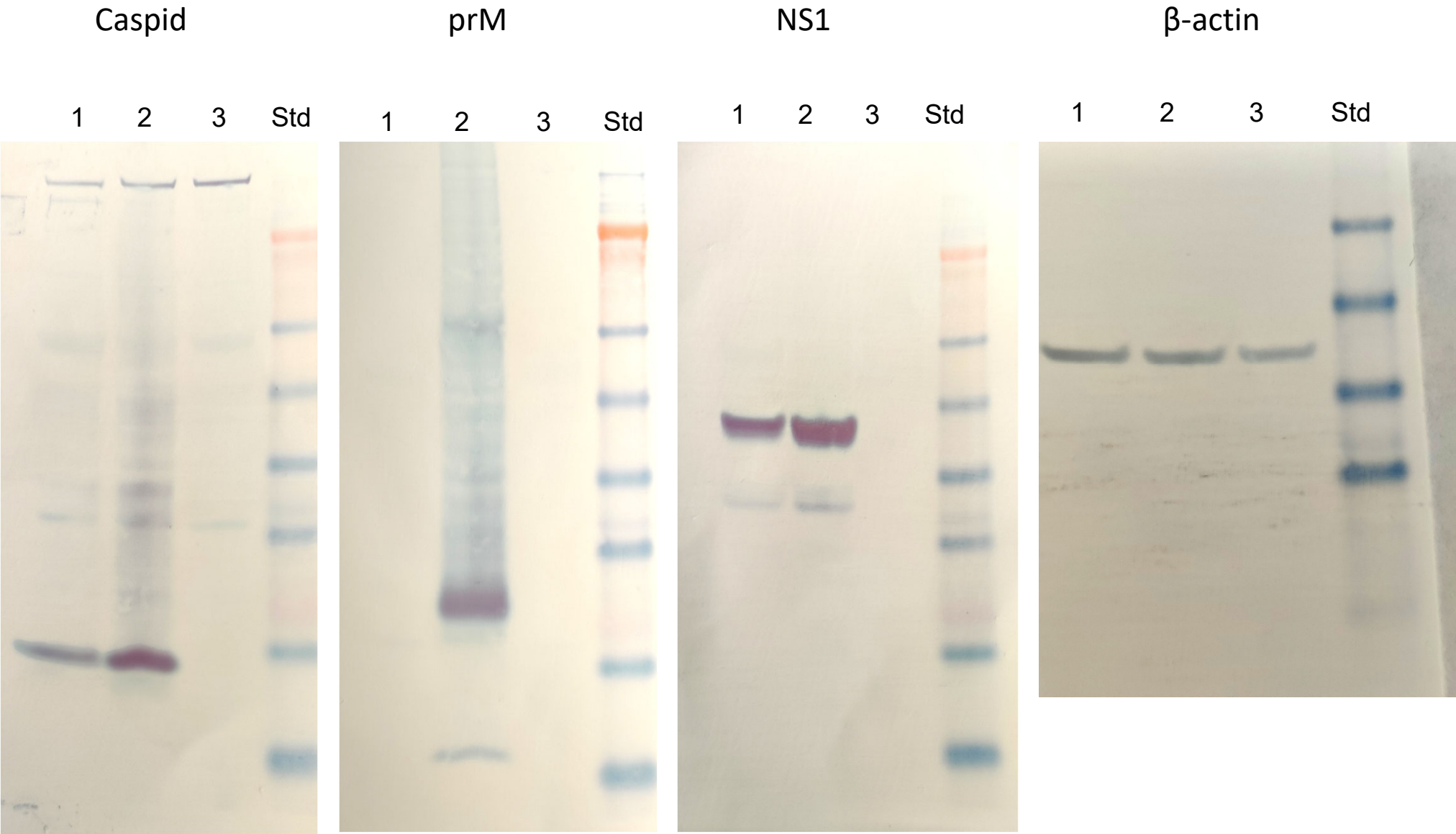

Figure 3A

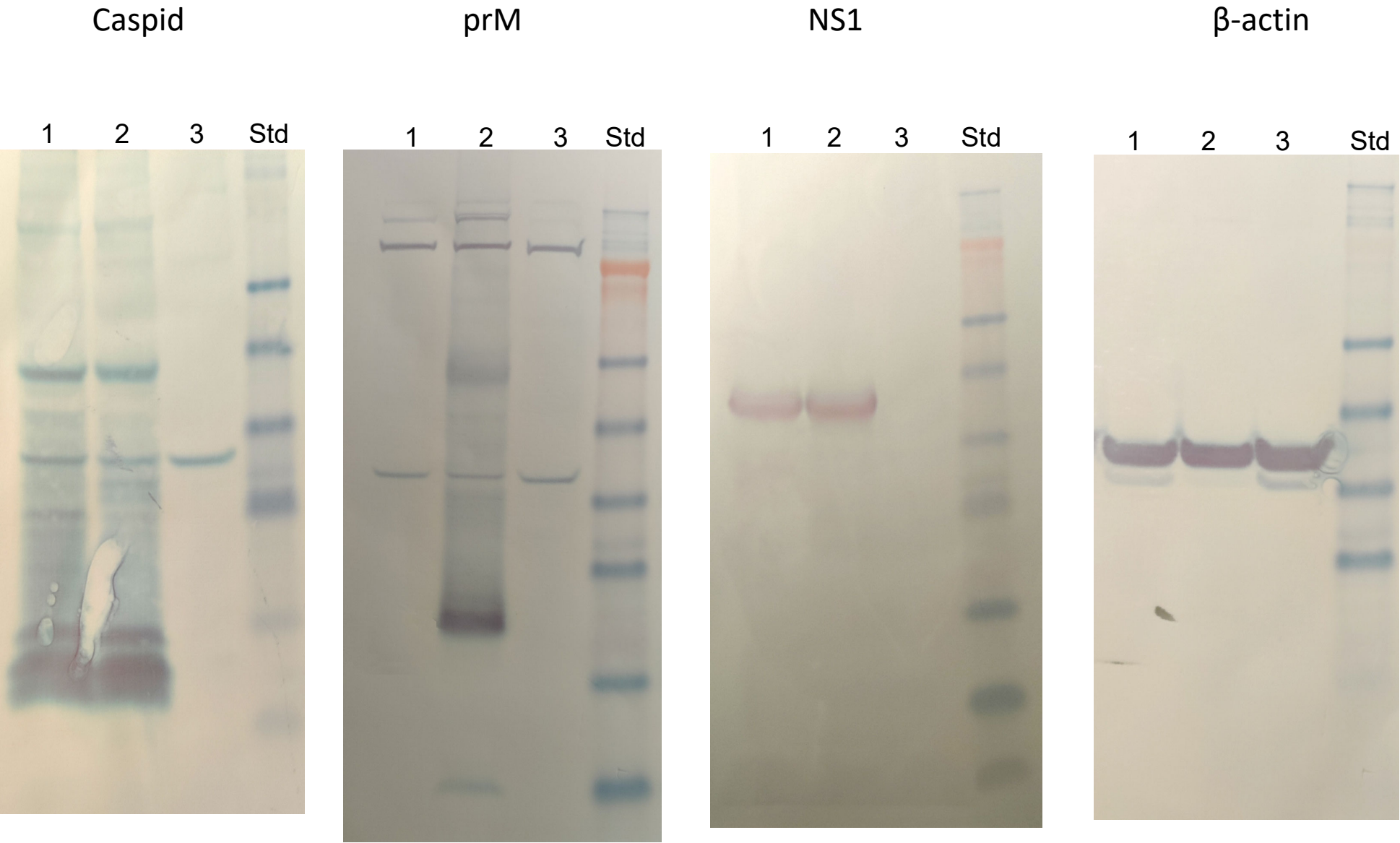

Supplement: Supplementary file 1 — Additional file 1. Figure S1. Mosquito and vertebrate cells support the replication of ZIKV/THOV(prM-E). A Western blot analysis of C/36 cells inoculated with ZIKV/THOV(prM-E). These are the same images as shown in Figure 2A, except they are uncropped. Experimental details are provided in the legend for Figure 2A. B Western blot analysis of Vero cells inoculated with ZIKV/THOV(prM-E). These are the same images as shown in Figure 3A, except they are uncropped. Experimental details can provided in the legend for Figure 3A [file 12985_2023_2172_MOESM1_ESM.pdf]
